# To what extent could eliminating racial discrimination reduce inequities in mental health and sleep problems among Aboriginal and Torres Strait Islander children? A causal mediation study

**DOI:** 10.1016/j.lanwpc.2024.101196

**Published:** 2024-10-08

**Authors:** Naomi Priest, Shuaijun Guo, Rushani Wijesuriya, Catherine Chamberlain, Rosemary Smith, Sharon Davis, Janine Mohamed, Margarita Moreno-Betancur

**Affiliations:** a The Centre for Social Policy Research, Australian National University, Canberra, Australia; b Centre for Community Child Health, Murdoch Children's Research Institute, Royal Children's Hospital, Melbourne, Australia; c Indigenous Health Equity Unit, Melbourne School of Population and Global Health, The University of Melbourne, Melbourne, Australia; d Department of Pediatrics, University of Melbourne, Melbourne, Australia; e Clinical Epidemiology and Biostatistics Unit, Murdoch Children's Research Institute, Royal Children's Hospital, Melbourne, Australia; f Judith Lumley Centre, La Trobe University, Melbourne, Australia; g The Lowitja Institute, Carlton, Australia; h NGANGK YIRA: Murdoch University Research Centre for Aboriginal Health and Social Equity, Murdoch, Australia; i Goorlil Consulting, Canberra, Australia

**Keywords:** Racism, Mental health, Sleep, Indigenous, Children, Life course

## Abstract

**Background:**

Racism is a fundamental cause of health inequities for Aboriginal and Torres Strait Islander children. We estimated the potential reduction in inequities in Aboriginal and Torres Strait Islander children's mental health and sleep problems if interpersonal racial discrimination was eliminated.

**Methods:**

We drew on cross-sectional data from the Speak Out Against Racism (SOAR; N = 2818) and longitudinal data from the Longitudinal Study of Australian Children (LSAC; N = 8627). The SOAR was completed in 2017 and the LSAC followed children from 2004 to 2014 in the kindergarten cohort and from 2008 to 2018 in the birth cohort. Exposure: Aboriginal and Torres Strait Islander status (Aboriginal and Torres Strait Islander/Anglo-European), a proxy measure of structural racism (SOAR: 10–15 years; LSAC: 4–5 years); Mediator: interpersonal racial discrimination (yes/no) (SOAR: 10–15 years; LSAC: 12–13 years); Outcomes: mental health problems (yes/no) and sleep problems (yes/no) (SOAR: 10–15 years; LSAC: 14–15 years). An interventional effects causal mediation approach was used.

**Findings:**

Aboriginal and Torres Strait Islander children had higher prevalence of mental health problems (SOAR: 40.1% versus 13.5%; LSAC: 25.3% versus 7.6%) and sleep problems (SOAR: 28.5% versus 18.4%; LSAC: 14.0% versus 9.9%) than Anglo-European children. Hypothetical interventions eliminating Aboriginal and Torres Strait Islander children's experiences of interpersonal racial discrimination could reduce 42.4% and 48.5% of mental health and sleep inequities in SOAR (equivalent to 11.2% and 4.7% absolute reductions) and 25.6% and 1.6% of mental health and sleep inequities in LSAC (equivalent to 5.5% and 0.1% absolute reductions). Absolute remaining inequities were similar across both studies for both outcomes.

**Interpretation:**

Targeted policy interventions that eliminate racial discrimination against Aboriginal and Torres Strait Islander children could have high potential to reduce inequities in mental health and sleep problems. Addressing racism and racial discrimination needs a multi-component and multi-level approach directed by Aboriginal and Torres Strait Islander communities.

**Funding:**

10.13039/501100000925National Health and Medical Research Council of Australia and Medical Research Future Fund of Australia.

**Research in context**

**Evidence before this study**

We searched four databases (Medline, PsycINFO, PubMed, and ERIC) via The University of Melbourne Library on 05, October 2023, for all publications since inception that used the interventional effects approach to quantify the extent to which intervening on racial discrimination could reduce disparities in health and wellbeing outcomes between Indigenous and non-Indigenous populations worldwide. This search did not identify any published studies, so we broadened our search to include studies with any population using a refined list of search terms (“racism”) AND ((“interventional effects”) OR (“causal mediation”)) AND ((“health”) OR (“wellbeing”)). This search yielded a total of six studies, with all studies using causal mediation analysis to investigate racial and ethnic disparities (e.g., Black-White, Asian-White, Hispanic-White) in a range of health outcomes including mortality, preterm birth, substance misuse, and dementia among US adults. Mediators examined included hospital type, maternal cardiometabolic risk factors, area deprivation index, psychological distress, racial discrimination in medical settings, and systemic inflammation, with the proportion mediated ranging from 1.5% to 65.8%. However, no studies were identified that investigated the role of interpersonal racial discrimination in mediating racial and ethnic disparities in health among children.

**Added value of this study**

This study used an interventional effects approach to estimate the extent to which intervening on interpersonal racial discrimination could reduce inequities in mental health and sleep problems among Aboriginal and Torres Strait Islander children. We used data from two studies–Speak Out Against Racism (SOAR) and the Longitudinal Study of Australian Children (LSAC)–to examine our research question, allowing us to explore common developmental associations within studies with complementary strengths across measures, sampling strategies and designs. We found larger inequalities present at the outset in SOAR compared to LSAC. In SOAR, we found that eliminating everyday experiences of racial discrimination could reduce almost half of the inequities in mental health (42.4%) and sleep difficulties (48.5%) experienced by Aboriginal and Torres Strait Islander children compared to their Anglo-European peers, while in LSAC, the reductions were lower (25.6% and 1.6% respectively). Absolute remaining inequities were similar across both studies for both outcomes indicating that addressing interpersonal racial discrimination is necessary but insufficient to eliminate inequities in mental health and sleep problems among Aboriginal and Torres Strait Islander children. Addressing the wider system and structure of racism as a fundamental cause of these inequities, and of which interpersonal racial discrimination is only one expression, is urgently required.

**Implications of all the available evidence**

Our findings confirm the well-established relationship between exposure to racism and poor mental health and sleep problems among Aboriginal and Torres Strait Islander children. The benefit observed across studies with complementary strengths suggests that targeted policy interventions that eliminate interpersonal racial discrimination have high potential to reduce inequities in mental health and sleep problems experienced by Aboriginal and Torres Strait Islander children. Critically, interpersonal racial discrimination is only one expression of the wider system of racism that impacts Aboriginal and Torres Strait Islander children. Multi-component and multi-level anti-racism actions directed by Aboriginal and Torres Strait Islander communities are needed to address racism in all its forms to achieve health equity and to realize fundamental human rights.

## Introduction

For generations the health and wellbeing of children has been nurtured by Aboriginal and Torres Strait Islander peoples through strong cultures, languages and systems of kinship and community care.^1^ Evidence shows that Aboriginal and Torres Strait Islander children were likely physically, socially and emotionally healthier than most European children at the time of colonization in 1788.^2^ With colonization, racism arrived in Australia, Aboriginal and Torres Strait Islander peoples were racialized and dehumanized by settler-colonists, who considered them a culturally inferior and primitive race to legitimize and excuse systemic violence, genocide, forced removal from lands and suppression of cultural practices.^3^ Current differences seen across a range of social and health indicators between Aboriginal and Torres Strait Islander peoples and non-Indigenous people are because of historical and ongoing effects of settler-colonialism and racism, rather than biology or race.^4^^,^^5^

Racism is an ideology and system of oppression that categorises and stratifies social groups into ‘races’ and then devalues and disadvantages those groups considered inferior and advantages those considered superior, differentially allocating to them valued societal power, resources and opportunities.^6^ Structural racism is the interconnected system of racialized rules, laws, policies, and practices underpinning and embedded in institutions such as health, education, justice, housing, banking, and the media.^7^ Considered by some as embedded within, and a crucial element of structural racism,^8^ and as distinct by others,^9^ cultural and ideological racism contribute to, and reinforce structural racism, with societal values and beliefs privileging and protecting Whiteness and White social and economic power^10^ and rationalizing and informing an uneven distribution of economic, political and social and even psychological rewards and resources along racialized lines.^8^ This includes framing populations as inferior or vulnerable in order to justify policies and systems that reinforce and embed inequities,^8^ for example in Australia the historical and contemporary policies of forced removal of Aboriginal and Torres Strait Islander children from their families (the ‘Stolen Generations’).^5^

Structural racism impacts multiple factors that lead to socioeconomic inequities, including access to education and employment, financial security, housing conditions, and neighbourhood quality and safety. Directly and indirectly, including through socioeconomic pathways, structural racism influences exposure to physical, psychosocial, and environmental stressors, which can in turn affect risks for poor physical and mental health, social and emotional wellbeing, and unhealthy behaviors (e.g., tobacco and alcohol use, physical inactivity).^11^

Structural racism is also expressed as interpersonal racial discrimination, an everyday form of racism occurring between individuals. Interpersonal racial discrimination enacts structural racism and racist ideologies - and is itself a powerful stressor and contributor to poor health and health inequities through psychological, behavioral and biological pathways.^5^^,^^11^ Aboriginal and Torres Strait Islander peoples have long said that interpersonal racial discrimination has harmful health consequences. Findings from systematic reviews and meta-analyses support this claim, with strong empirical evidence of negative health effects throughout life.^12^^,^^13^

Children are particularly sensitive to the negative effects of interpersonal racial discrimination with childhood developmentally, biologically and socially significant.^14^ Childhood sets the foundation for lifelong health and wellbeing with many chronic conditions including mental illness^15^ shown to have early life origins. Childhood experiences of interpersonal racial discrimination are associated with negative effects on health in childhood and adulthood.^12^^,^^16^ Extant evidence among Aboriginal and Torres Strait Islander children aligns with international findings, documenting associations between childhood interpersonal racial discrimination and poor health, including mental health and sleep difficulties.^17^ However, the extent to which intervening on racial discrimination would reduce inequities in health experienced by Aboriginal and Torres Strait Islander children compared to their non-Indigenous peers remains unknown. To our knowledge, no study internationally has estimated the extent to which intervening on racial discrimination could reduce inequities in Indigenous child health. Eliminating racism is a longstanding priority for Aboriginal and Torres Strait Islander peoples. Although addressing racism as a critical public health threat is increasingly recognized internationally,^18^ including for children,^19^ progress remains slow.

In this study, we aimed to quantify the potential benefit of intervening to eliminate interpersonal racial discrimination experiences among Aboriginal and Torres Strait Islander children to reduce inequities in mental health and sleep problems. We applied causal mediation analysis based on the interventional effects approach—a recently developed statistical approach to evaluate hypothetical interventions.^20^ We focused on inequities in mental health problems, the leading disease group contributing to disease burden in the Aboriginal and Torres Strait Islander population. The study was collaboratively developed between Aboriginal and non-Indigenous researchers, from formation of research questions through to interpretation and discussion of findings.

## Methods

### Data sources

We drew on both cross-sectional and longitudinal data from two Australian large-scale population surveys to address the research question. Our purpose in considering these two studies was to examine our research question in two independent samples, allowing us to explore common developmental associations across studies with complementary strengths across designs, sampling strategies and measures.^21^

#### Speak Out Against Racism (SOAR)

SOAR is a large-scale population-representative cross-sectional study on racism and mental health that was completed by 4664 primary and secondary students aged 10–15 years across 23 schools in New South Wales and Victoria in 2017.^22^ In brief, a stratified sampling method balanced on the available school characteristics was used to ensure the sample was representative of the overall school population in NSW and Victoria, with an oversampling of schools with higher proportions of Aboriginal and Torres Strait Islander students. Ethics approval was obtained from the Australian National University and from each state government education department, and permission was obtained from each participating school principal, with parent opt-out consent and student assent.

#### Growing up in Australia: the Longitudinal Study of Australian Children (LSAC)

LSAC is a nationally representative study comprised of two cohorts of Australian children: a birth cohort (B-cohort) of 5107 infants; and a kindergarten cohort (K-cohort) of 4983 four-year-olds. The study commenced in May 2004. In short, a two-stage clustered design was employed, where first the postcodes (i.e. clusters) were randomly sampled, followed by randomly sampling children within each cluster, to select a sample that was broadly representative of the Australian child population except those living in remote areas (as some remote postcodes were excluded in stage one due to high cost of data collection).^23^ To ensure that meaningful associations for Aboriginal and Torres Strait Islander children could be estimated with adequate precision, we drew on aligned data from both the B-cohort and K-cohort (see Supplementary file 1). LSAC was approved by the Australian Institute of Family Studies Human Research Ethics Review Board (ID 13-04).

### Measures

**Fig. 1:**
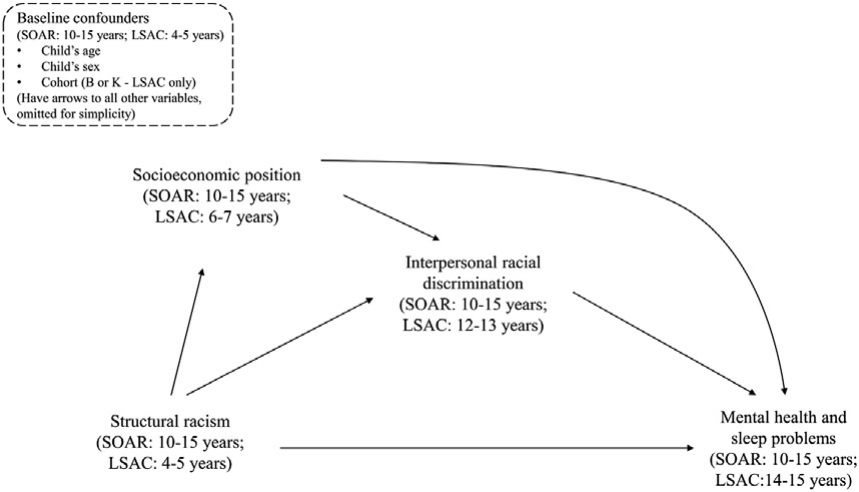
Conceptual model that depicts the relationship between Aboriginal and Torres Strait Islander status (as proxy for structural racism exposure rather than representing race and ethnicity as biological or innate constructs) and mental health and sleep problems via interpersonal racial discrimination.

Our conceptual model shown in Fig. 1 visually represents the hypothesized causal processes from structural racism to children's mental health and sleep problems, informed by current knowledge (see Supplementary file 2). This model depicts the pathways from Aboriginal and/or Torres Strait Islander status—as a proxy for structural racism and associated exposures, not as an indicator of innate, biological vulnerability^24^– to mental health and sleep problems, via interpersonal racial discrimination as the intervention target of interest. Fig. 1 was used to guide the selection of variables (Table 1) and inform the analytic approach.

**Table 1:** Measurement of exposure, mediator, outcomes and potential confounders.

Variable	Measurement details	
	SOAR	LSAC
**Exposure**		
Structural racism	Aboriginal and Torres Strait Islander status and ethnicity in SOAR were captured at 10–15 years using a self-reported measure with categories developed for the study, given ethnicity beyond Aboriginal and Torres Strait Islander status is not routinely collected in Australia.^22^ In this study, the SOAR sample was restricted to those from Aboriginal and Torres Strait Islander background (n = 409) and Anglo-European backgrounds (n = 2409).	Aboriginal and Torres Strait Islander and ethnicity in LSAC were derived from three indicators reported by the child's primary carer at 4–5 years: country of birth (both child and parents), language spoken at home (both child and parents), and Aboriginal and Torres Strait Islander status (child). We created three mutually exclusive categories: Aboriginal and/or Torres Strait Islander; ethnic minority (non-White and not Aboriginal and/or Torres Strait Islander) and Anglo-European following previous approaches.^25^ Similarly, we restricted the LSAC sample to those from Aboriginal and Torres Strait Islander (n = 417) and Anglo-European backgrounds (n = 8210).
**Mediator**		
Interpersonal racial discrimination	Direct racial discrimination in SOAR was measured at 10–15 years using 10 items drawn from the Adolescent Discrimination Distress Index (ADDI) together with two items used previously with diverse Australian school students.^17^ Students responded to each item as ‘this did not happen to me’, ‘once or twice’, ‘every few weeks’, ‘about once a week’, ‘several times a week or more’. A binary response (‘This did not happen to me’ versus ‘once or twice/every few weeks/about once a week/several times a week or more’) was created for each item. Students who answered ‘yes’ to any of the twelve items were considered as ‘having ever experienced racial discrimination’.	Direct racial discrimination in LSAC was measured using three questions reported by the study child at 12–13 years. Children were asked whether in the last six months they had been treated unfairly or badly (‘yes’ or ‘no’) because of their language or accent, skin color, or cultural backgrounds. Children who answered ‘yes’ to any of the three questions were considered as ‘having ever experienced racial discrimination’.
**Outcomes**		
Mental health problems	Children's mental health problems were assessed using the Strengths and Difficulties Questionnaire (SDQ), which is a brief validated screening measure of behavioral and emotional problems for 3–16 year olds. The SDQ total score of socioemotional difficulties is a sum of scores on 20 items, with higher scores representing poorer mental health. Student-report SDQ ratings at 10–15 years were used in SOAR. Given that the SDQ is not conceptually equivalent between Aboriginal and Torres Strait Islander children and non-Indigenous children,^26^ Aboriginal and Torres Strait Islander children were coded as having elevated mental health symptoms if they scored 16–40 and non-Indigenous children if they scored 20–40.	The SDQ was also used in LSAC to measure children's mental health problems. Child-report SDQ ratings at 14–15 years were used in LSAC. Aboriginal and Torres Strait Islander children were coded as having elevated mental health symptoms if they scored 16–40 and non-Indigenous children if they scored 20–40.
Sleep problems	Sleep problems at 10–15 years in SOAR were measured using the item “During the past four weeks, how often did you awake during your sleep time and have trouble falling back to sleep again?”, coded as “no” (none/a little, some/a good bit of the time) versus “yes” (most/all of the time).	Sleep problems at 14–15 years in LSAC were measured using the item “During the last month, how well do you feel you have slept in general?”, with sleep problems coded as “no” (very well/fairly well) versus “yes” (fairly badly/very badly).
**Confounders**		
Baseline confounders	Two baseline confounders at 10–15 years were measured in SOAR: child's age (continuous) and child's sex (male/female).	Two baseline confounders at 4–5 years were measured in LSAC: child's age (continuous) and child's sex (male/female), and cohort (birth (B) cohort/kindergarten (K) cohort).
Intermediate confounder	Socioeconomic position was treated as an intermediate confounder, given it is affected by structural racism. Socioeconomic position at 10–15 years in SOAR was measured at the school level using the Index of Community Socio-Educational Advantage. In keeping with previous studies,^27^ we used the bottom 25th percentile to indicate low socioeconomic position.	Socioeconomic position at 6–7 years in LSAC was measured at the family level using a composite of each parent's self-reported annual income, highest education and occupation level. Following previous approaches,^27^ socioeconomic position was dichotomized using the bottom 25th percentile to indicate low socioeconomic position. We also conducted a sensitivity analysis in LSAC, where there was broader data available, including five additional intermediate confounders at 6–7 years (household member mental health, household member substance use, family violence, child disability and remoteness) (see Supplementary file 4 for details).

LSAC, Longitudinal Study of Australian Children; SOAR, Speak Out Against Racism.

### Statistical analysis

Descriptive statistics were calculated for all study variables in the total sample and stratified by Aboriginal and Torres Strait Islander status. Preliminary analyses were then conducted to examine the pathways depicted in Fig. 1. We used generalized estimating equations (GEE) to account for the cluster correlations due to schools (SOAR) and postcodes (LSAC) in the data. Specifically, GEEs with log-Poisson link and an exchangeable correlation matrix were used to estimate risk ratios representing the unadjusted and adjusted (controlling for child's age, child's sex, and cohort design (LSAC only)) associations between Aboriginal and Torres Strait Islander status and each outcome, between Aboriginal and Torres Strait Islander status and racial discrimination, and between racial discrimination and each outcome. An exchangeable correlation structure was chosen for the working correlation structure in GEEs,^28^ as it was reasonable to assume that pairs of units within clusters (schools and postcodes) were exchangeable. Descriptive statistics and preliminary analyses were conducted using Stata 18.0.

#### Causal mediation analysis

For each outcome, we conducted a causal mediation analysis using an interventional effects approach to estimate (1) the overall inequities in the prevalence of each outcome between Aboriginal and Torres Strait Islander children and Anglo-European children, and (2) the benefit (in terms of reducing outcome prevalence) of a hypothetical intervention eliminating racial discrimination (i.e. a maximum benefit intervention scenario) in Aboriginal and Torres Strait Islander children. For this purpose, we followed the framework outlined by Moreno-Betancur et al.,^20^ defining interventional mediation effects that map to a target trial, i.e. the randomized trial that would have been ideally conducted to assess the impact of such a hypothetical intervention. Analyses using interventional effects are increasingly used in health inequities research and are suited to contexts where data on actual, well-defined interventions already implemented in the community are not available.^29^

The interventional effects of interest were then estimated using an extended g-computation estimation procedure, which uses a series of richly specified (with interactions) regression models for any intermediate confounders, the mediator and the outcome (see Supplementary file 3). The primary analysis included only one intermediate confounder socioeconomic position, at 10–15 years and 6–7 years in SOAR and LSAC, respectively. We also conducted a sensitivity analysis in LSAC, where there was broader data available, including five additional intermediate confounders at 6–7 years (household member mental health, household member substance use, family violence, child disability, and remoteness) (see Supplementary file 4 for details).

The overall inequities (with no mediator intervention) were estimated by comparing the outcome prevalence under exposure and no exposure, estimated using predictions from the relevant outcome model. The prevalence of each outcome in Aboriginal and Torres Strait Islander children under an intervention that would completely eliminate racial discrimination was estimated by using the series of intermediate confounder, mediator and outcome models to predict outcomes when setting the value of the mediator under exposure to zero (no racial discrimination). From this, we could estimate the reduction in prevalence of each outcome in Aboriginal and Torres Strait Islander children, and the inequities that would remain after intervening to eliminate racial discrimination. Standard errors for all estimates were computed using a clustered bootstrap procedure. All causal mediation analyses were implemented using R Statistical Software 4.2.2.

#### Missing data

The overall proportion of children with missing data in any of the study variables was 12.1% and 41.4% in SOAR and LSAC, respectively. Multiple imputation by chained equations was used in each study to reduce bias due to incomplete records for preliminary and causal mediation analyses. We imputed continuous and binary variables using univariate linear and logistic regression models respectively, using Stata 18.0. The imputation models included all study variables as well as all two-way interactions amongst exposure, mediator, outcome, baseline and intermediate confounders to ensure compatibility with the regression models used in extended g-computation for the causal mediation analysis (see Supplementary file 3). Clustering by school in SOAR and by residential postcodes in LSAC was accounted for in the imputation procedure by including a categorical cluster membership variable as a predictor in the univariate imputation models. Based on the percentage of missing data, we imputed twenty datasets for SOAR and fifty for LSAC. Post-imputation, the analyses were conducted in each of the imputed datasets and the results were combined using Rubin's rules to obtain the final estimates of the parameters of interest.

### Role of funding sources

No funders had any role in study design, data analysis, interpretation, preparation of the manuscript and decision to submit for this present study. This present study analysed secondary data and did not gather any new data.

## Results

### Sample characteristics

Participant characteristics are summarized in Table 2. SOAR had a higher proportion of Aboriginal and Torres Strait Islander children than LSAC (14.5% versus 4.8%). Around half (50.1%) of Aboriginal and Torres Strait Islander children in SOAR experienced racial discrimination and 26.0% in LSAC. At outcome assessment, Aboriginal and Torres Strait Islander children in SOAR had higher prevalence of elevated mental health symptoms (40.1% versus 25.3%) and sleep problems (28.5% versus 14.0%) than those in LSAC.

**Table 2:** Descriptive information for all study variables in SOAR and LSAC samples. Observed data are shown.

Variable	SOAR (N = 2818)				LSAC (N = 8627)			
	Frequency (%)/Mean (±SD)	Aboriginal and Torres Strait Islander status		Missing n (%)	Frequency (%)/Mean (±SD)	Aboriginal and Torres Strait Islander status		Missing n (%)
		No	Yes			No	Yes	
**Exposure**								
Aboriginal status				0				0
No	2409 (85.5)	–	–		8210 (95.2)	–	–	
Yes	409 (14.5)	–	–		417 (4.8)	–	–	
**Mediator**								
Racial discrimination				83 (2.9)				2460 (28.5)
No	1877 (68.6)	1680 (71.8)	197 (49.9)		5665 (91.9)	5531 (92.4)	134 (74.0)	
Yes	858 (31.4)	660 (28.2)	198 (50.1)		502 (8.1)	455 (7.6)	47 (26.0)	
**Outcome**								
Elevated mental health symptoms				56 (2.0)				3072 (35.6)
No	2282 (82.6)	2043 (86.5)	239 (59.9)		5109 (92.0)	5000 (92.4)	109 (74.7)	
Yes	480 (17.4)	320 (13.5)	160 (40.1)		446 (8.0)	409 (7.6)	37 (25.3)	
Sleep problems				230 (8.2)				3081 (35.7)
No	2075 (80.2)	1819 (81.6)	256 (71.5)		4993 (90.0)	4870 (90.1)	123 (86.0)	
Yes	513 (19.8)	411 (18.4)	102 (28.5)		553 (10.0)	533 (9.9)	20 (14.0)	
**Baseline confounders**								
Child's age at baseline	12.35 (1.48)	12.40 (1.47)	12.04 (1.50)	67 (2.4)	4.21 (0.41)	4.21 (0.41)	4.25 (0.43)	575 (6.7)
Child's sex				79 (2.8)				0
Male	1359 (49.6)	1144 (48.7)	215 (55.3)		4393 (50.9)	4181 (50.9)	212 (50.8)	
Female	1380 (50.4)	1206 (51.3)	174 (44.7)		4234 (49.1)	4029 (49.1)	205 (49.2)	
Cohort				–				0
B-cohort	–	–	–		4407 (51.1)	4177 (50.9)	230 (55.2)	
K-cohort	–	–	–		4220 (48.9)	4033 (49.1)	187 (44.8)	
**Intermediate confounder**								
Socioeconomic position				0				1113 (12.9)
Top 75%–Middle or high	2066 (73.3)	1848 (76.7)	218 (53.3)		5635 (75.0)	5511 (76.3)	124 (42.9)	
Bottom 25% - Low	752 (26.7)	561 (23.3)	191 (46.7)		1879 (25.0)	1714 (23.7)	165 (57.1)	

B-cohort, Birth Cohort; K-cohort, Kindergarten Cohort; LSAC, Longitudinal Study of Australian Children; SD, Standard Deviation; SOAR, Speak Out Against Racism.

### Associations between Aboriginal and Torres Strait Islander status, racial discrimination, and mental health and sleep problems

Aboriginal and Torres Strait Islander children had higher risk of experiencing racial discrimination (Table 3; SOAR: RR = 1.69, 95% CI: 1.47–1.95; LSAC: RR = 3.64, 95% CI: 2.88–4.61), elevated mental health symptoms (SOAR: RR = 2.89, 95% CI: 2.27–3.69; LSAC: RR = 3.68, 95% CI: 2.79–4.86) and sleep problems (SOAR: RR = 1.42, 95% CI: 1.09–1.85; LSAC: RR = 1.57, 95% CI: 1.07–2.32) than Anglo-European children, after adjusting for sex, age, and cohort (LSAC only). Children who experienced racial discrimination had higher risk of elevated mental health symptoms (SOAR: RR = 2.19, 95% CI: 1.81–2.66; LSAC: RR = 2.21, 95% CI: 1.70–2.87) and sleep problems (SOAR: RR = 1.58, 95% CI: 1.35–1.84; LSAC: RR = 1.59, 95% CI: 1.21–2.08) than those who did not, after adjusting for sex, age, cohort (LSAC only), Aboriginal and Torres Strait Islander status, and socioeconomic position. These findings provide support for the detrimental effect of racial discrimination on children's mental health and sleep problems.

**Table 3:** Generalized estimating equations examining the associations between Aboriginal status, racial discrimination, and mental health and sleep problems, based on multiple imputation in each study (SOAR, N = 2818 and LSAC, N = 8627).

Models	SOAR (RR and its 95% CI)		LSAC (RR and its 95% CI)	
	Unadjusted	Adjusted^a^	Unadjusted	Adjusted^a^
**Association with elevated mental health symptoms**				
Aboriginal and Torres Strait Islander status (Ref = no)	2.87 (2.24, 3.68)	2.89 (2.27, 3.69)	3.69 (2.79, 4.89)	3.68 (2.79, 4.86)
Racial discrimination (Ref = no)	2.46 (2.02, 3.00)	2.19 (1.81, 2.66)	2.73 (2.12, 3.51)	2.21 (1.70, 2.87)
**Association with sleep problems**				
Aboriginal and Torres Strait Islander status (Ref = no)	1.40 (1.08, 1.82)	1.42 (1.09, 1.85)	1.58 (1.07, 2.34)	1.57 (1.07, 2.32)
Racial discrimination (Ref = no)	1.60 (1.36, 1.88)	1.58 (1.35, 1.84)	1.62 (1.24, 2.11)	1.59 (1.21, 2.08)
**Association with racial discrimination**				
Aboriginal and Torres Strait Islander status (Ref = no)	1.72 (1.49, 1.98)	1.69 (1.47, 1.95)	3.63 (2.87, 4.59)	3.64 (2.88, 4.61)

CI, confidence interval; LSAC, Longitudinal Study of Australian Children; Ref, reference group; RR, risk ratio; SOAR, Speak Out Against Racism.

a Confounders adjusted for were child's age, sex, and cohort (B or K–LSAC only). Models for the association between racial discrimination and each outcome were additionally adjusted for Aboriginal and Torres Strait Islander status and socioeconomic position.

### The extent to which a hypothetical intervention eliminating racial discrimination reduces inequities

Table 4 displays the results from the causal mediation analysis using the interventional effects approach. In terms of the overall inequities in each outcome between Aboriginal and Torres Strait Islander children and their Anglo-European peers, we found that the absolute difference in the prevalence of elevated mental health symptoms was 26.4% (95% CI: 21.4%–31.3%) in SOAR, adjusted for age and sex, while for sleep problems it was 9.7% (95% CI: 3.3%–16.1%). In LSAC, the absolute difference was 21.5% (95% CI: 13.8%, 29.1%) in the prevalence of elevated mental health symptoms and 6.1% (95% CI: −0.2% to 12.4%) in sleep problems for Aboriginal and Torres Strait Islander children compared with their Anglo-European peers, after adjusting for age, sex, and cohort.

**Table 4:** Results from causal mediation analysis: estimated effects on the prevalence of mental health and sleep problems by hypothetical interventions eliminating racial discrimination in Aboriginal and Torres Strait Islander children, using multiply imputed data for the full cohorts in SOAR and LSAC.

Effect	SOAR (N = 2818)			LSAC (N = 8627)		
	Estimate of absolute risk difference (%) 95% CI	Proportion of inequities eliminated	Remaining inequities (%) 95% CI	Estimate of absolute risk difference (%) 95% CI	Proportion of inequities eliminated	Remaining inequities (%) 95% CI
**Mental health problems**						
Overall inequities	26.4 (21.4, 31.3)	–	–	21.5 (13.8, 29.1)	–	–
Reduction from intervening on racial discrimination	11.2 (5.6, 16.8)	42.4	15.2 (9.0, 21.3)	5.5 (−0.1, 11.1)	25.6	16.0 (7.4, 24.5)
**Sleep problems**						
Overall inequities	9.7 (3.3, 16.1)	–	–	6.1 (−0.2, 12.4)	–	–
Reduction from intervening on racial discrimination	4.7 (2.7, 9.2)	48.5	5.0 (0, 12.0)	0.1 (−4.7, 4.9)	1.6	6.0 (−1.4, 13.4)

CI, confidence interval; LSAC, Longitudinal Study of Australian Children; SOAR, Speak Out Against Racism. All estimates are adjusted. Baseline confounders controlled for were child's age, sex, and cohort (B or K–LSAC only). Intermediate confounder controlled for was socioeconomic position.

In SOAR, we estimated that a hypothetical intervention eliminating racial discrimination experienced by Aboriginal and Torres Strait Islander children would result in an 11.2% (95% CI: 5.6%–16.8%) and 4.7% (95% CI: 2.7%–9.2%) absolute reduction in the prevalence of mental health and sleep problems respectively in Aboriginal and Torres Strait Islander children. These estimates correspond to 42.4% and 48.5% inequities eliminated in mental health and sleep problems respectively. In LSAC, we found that a hypothetical intervention eliminating racial discrimination experienced by Aboriginal and Torres Strait Islander children would result in a 5.5% (95% CI: −0.1% to 11.1%) and 0.1% (95% CI: −4.7%, 4.9%) absolute reduction in the prevalence of mental health and sleep problems respectively, corresponding to 25.6% and 1.6% inequities eliminated in mental health and sleep problems. Similar and large inequities remain in both studies after the hypothetical intervention on racial discrimination. Estimates in LSAC remained largely unchanged when the causal mediation analysis approach also modelled the five additional intermediate confounders (see Supplementary file 4).

## Discussion

### Summary of key findings

Using studies with diverse strengths, we estimated the potential benefit of hypothetical interventions that would eliminate interpersonal racial discrimination to reduce inequities in mental health and sleep problems among Aboriginal and Torres Strait Islander children. Our findings confirmed the large existing inequities in mental health and sleep problems between Aboriginal and Torres Strait Islander children and their Anglo-European peers.^17^^,^^30^ Our estimates from both studies suggest substantial potential benefits of eliminating racial discrimination to reduce mental health inequities among Aboriginal and Torres Strait Islander children; we also observed substantial benefits in reducing inequities in sleep problems in SOAR. Remaining inequities were similar across both studies for each outcome.

We found that the potential benefit of eliminating experiences of Aboriginal and Torres Strait Islander children's racial discrimination appeared to be more prominent in SOAR than in LSAC. There were also larger initial inequities observed before the hypothetical intervention in SOAR than in LSAC. Aside from the variation in study design (cross-sectional and longitudinal, respectively), variation in sampling strategies and measurement instruments may explain these differences. First, the sample in SOAR was based on an oversampling of schools with higher proportions of Aboriginal and Torres Strait Islander students, so that it had a more representative sample of Aboriginal and Torres Strait Islander children than LSAC. Second, SOAR used a 12-item instrument to measure interpersonal racial discrimination that captured a broad range of experiences with five response categories for each item, whereas LSAC asked children to answer ‘yes/no’ using three general items (“because of their language or accent, skin color, or cultural backgrounds”) regarding racial discrimination. Further studies that combine the strengths of SOAR and LSAC are needed to replicate our findings.

Our findings, that eliminating interpersonal racial discrimination had positive effects on Aboriginal and Torres Strait Islander children's mental health and sleep inequities, build on and are consistent with previous studies.^5^^,^^31^ Although to our knowledge there have been no studies globally that specifically quantify the extent to which intervening on interpersonal racial discrimination could reduce inequities in mental health and sleep problems among Indigenous children relative to non-Indigenous children, some studies addressed related questions. Using data from the Longitudinal Study of Indigenous Children, Shepherd et al.^31^ used the population attributable risk (PAR) as a measure of how much disease could be averted if interpersonal racial discrimination was eliminated. They found that racial discrimination accounted for 16.2% and 19.1% of the PAR for mental health problems and sleep difficulties respectively. Using population attributable fractions (PAFs), Thurber et al.^5^ recently showed that everyday racial discrimination could explain 47.4% of the overall gap in psychological distress between Indigenous and non-Indigenous adults. However, both PARs and PAFs do not estimate the reduction of inequalities in the risk of poor outcomes that can be achieved by intervening to eliminate racial discrimination.

We found that eliminating interpersonal racial discrimination could lead to large reductions in inequities in mental health and sleep difficulties experienced by Aboriginal and Torres Strait Islander children. Our findings add to current evidence regarding the substantial impact of racial discrimination on child health and child health inequities and reinforce calls to address racism and racial discrimination as a critical and urgent public health priority. To our knowledge, no other factor has been found to provide such a promising intervention target to reduce health inequities experienced by Aboriginal and Torres Strait Islander children. This further reinforces the substantial impact of racial discrimination on mental health inequities experienced by Aboriginal and Torres Strait Islander peoples. Urgent re-orientation of health policy, practice and research to address racism among Aboriginal and Torres Strait Islander children, their families and communities is required.

While the results suggest the potential benefit of eliminating racial discrimination, large inequities in mental health and sleep problems remain between Aboriginal and Torres Strait Islander children and their Anglo-European peers after the hypothetical intervention. Structural racism can impact health through a range of direct and indirect pathways, with interpersonal racial discrimination only one of these.^10^^,^^11^ Notwithstanding the critical importance of addressing interpersonal racial discrimination, further action is needed across multiple strategies to address structural racism and to fully achieve health equity and human rights. It can be suggested that if racism in all forms was eliminated from society, there would be no remaining health inequities experienced by Aboriginal and Torres Strait Islander children at all.

### Strengths and limitations

A key strength of this study is the replication of analyses in two representative samples of Australian children with complementary strengths in terms of design, measurement, and sampling, enhancing confidence of our findings. Second, we used emerging causal mediation methods that allow us to draw meaningful and policy-relevant insights from existing high-quality observational data. Nevertheless, there are several limitations. First, while we conducted multiple imputation to reduce the potential for selection bias due to missing data, biases might remain. Second, measurement error likely impacts our measures for structural racism and interpersonal racial discrimination. We rely on Aboriginal and Torres Strait Islander status as a proxy measure of structural racism, in the absence of additional measures that capture the complexity of social and institutional aspects of structural racism. Interpersonal racial discrimination measures used in the present study only capture a limited range of interpersonal racial discrimination experiences, particularly in LSAC. Therefore, the prevalence of interpersonal racial discrimination, and the potential benefits to mental health and sleep inequalities of reducing interpersonal racial discrimination were likely underestimated. Third, we were only able to evaluate the effects of a hypothetical, not actual, intervention. Finally, while we accounted for a host of baseline and intermediate confounders, unmeasured confounding and residual confounding due to measurement error in confounders might result in bias in our estimates.

### Implications and future directions

In a context where the COVID-19 pandemic and the Voice referendum in Australia have resulted in rising racism towards Aboriginal and Torres Strait Islander children and magnified the inequities they, their families and communities experience,^32^ actions to address racism and its impacts are urgent and essential. While our findings suggest eliminating interpersonal racial discrimination would have substantial benefits for reducing inequities in mental health and sleep problems among Aboriginal and Torres Strait Islander children, this study cannot address the practicalities of real-life implementation. Further research and policy efforts are needed to understand how existing policies, resources or actual anti-racism intervention programs in the real world can be leveraged to achieve the effects observed in this study.

Interpersonal racial discrimination is only the tip of the iceberg of the system of racism that impacts Aboriginal and Torres Strait Islander children, families and communities. Further attention should be given to upstream structural racism interventions within settings such as healthcare, housing, and employment, and addressing outstanding issues such as constitutional recognition and meeting human rights obligations.^33^ We suggest that in a world where there is no structural racism, there would be no gap at all between Aboriginal and Torres Strait Islander children and their non-Indigenous peers. Future efforts to address racism are needed and these should be driven by Aboriginal and Torres Strait Islander communities.

## Conclusions

We found that eliminating interpersonal racial discrimination could lead to large reductions in inequities in mental health and sleep difficulties experienced by Aboriginal and Torres Strait Islander children compared to their Anglo-European peers. However, we estimated that large inequities would remain, meaning attention to interpersonal racial discrimination without addressing structural racism may produce few gains. Continued policy and practice efforts driven by Aboriginal and Torres Strait Islander communities are needed to address racism and its negative impact on Aboriginal and Torres Strait Islander children.

## Contributors

NP conceptualized the initial idea for the study. NP, CC, RS, SD, JM, and MM-B refined the scope of the study. NP, SG, RW, and MM-B contributed to the data analysis plan. SG and RW have accessed and verified the underlying data. SG and RW conducted the data analysis. MM-B provided statistical advice. RW and MM-B cross-checked coding. SG did the literature review. NP and SG produced the initial draft of the manuscript. All authors were involved in interpreting results and drafting the manuscript. All authors approved the final version of the manuscript, take responsibility for its content, and were responsible for the decision to submit the manuscript.

## Data sharing statement

All data used in this manuscript are available on application to the Longitudinal Study of Australian Children (https://dataverse.ada.edu.au/dataverse/lsac) and from the corresponding author on request for the Speak Out Against Racism study.

## Declaration of interests

We declare no competing interests.
